# Inhibition of growth of hepatocellular carcinoma by co-delivery of anti-PD-1 antibody and sorafenib using biomimetic nano-platelets

**DOI:** 10.1186/s12885-024-12006-1

**Published:** 2024-02-26

**Authors:** Xuanbo Da, Bangping Cao, Jiantao Mo, Yukai Xiang, Hai Hu, Chen Qiu, Cheng Zhang, Beining Lv, Honglei Zhang, Chuanqi He, Yulong Yang

**Affiliations:** 1 Center of Gallbladder Disease, Shanghai East Hospital, Institute of Gallstone Disease, School of Medicine, Tongji University, 200092 Shanghai, China; 2 School and Hospital of Stomatology, Shanghai Engineering Research Center of Tooth Restoration and Regeneration, Tongji University, 200072 Shanghai, China; 3 Department of Hepatobiliary Surgery, First Affiliated Hospital of Xi’an Jiaotong University, 710061 Xi’an, Shaanxi China

**Keywords:** Platelet membrane, Immunotherapy, Sorafenib, Tumor microenvironment, Nanoparticles

## Abstract

**Background:**

Traditional nanodrug delivery systems have some limitations, such as eliciting immune responses and inaccuracy in targeting tumor microenvironments.

**Materials and methods:**

Targeted drugs (Sorafenib, Sora) nanometers (hollow mesoporous silicon, HMSN) were designed, and then coated with platelet membranes to form aPD-1-PLTM-HMSNs@Sora to enhance the precision of drug delivery systems to the tumor microenvironment, so that more effective immunotherapy was achieved.

**Results:**

These biomimetic nanoparticles were validated to have the same abilities as platelet membranes (PLTM), including evading the immune system. The successful coating of HMSNs@Sora with PLTM was corroborated by transmission electron microscopy (TEM), western blot and confocal laser microscopy. The affinity of aPD-1-PLTM-HMSNs@Sora to tumor cells was stronger than that of HMSNs@Sora. After drug-loaded particles were intravenously injected into hepatocellular carcinoma model mice, they were demonstrated to not only directly activate toxic T cells, but also increase the triggering release of Sora. The combination of targeted therapy and immunotherapy was found to be of gratifying antineoplastic function on inhibiting primary tumor growth.

**Conclusions:**

The aPD-1-PLTM-HMSNs@Sora nanocarriers that co-delivery of aPD-1 and Sorafenib integrates unique biomimetic properties and excellent targeting performance, and provides a neoteric idea for drug delivery of personalized therapy for primary hepatocellular carcinoma (HCC).

**Supplementary Information:**

The online version contains supplementary material available at 10.1186/s12885-024-12006-1.

## Background

According to global cancer Statistics 2018, the incidence and mortality rate of primary liver cancer ranks 6th and 4th among all malignancies respectively [1]. Although targeted therapy is thought as the main means of liver cancer treatment, it is difficult to cure tumors clinically as a result of the occurrence of tumor drug resistance. Immunotherapy has become a new method for the clinical therapy of primary hepatocellular carcinoma (HCC) recently. However, the objective response rate (ORR) of HCC patients is only 10-20% by only using immune checkpoint inhibitors (ICIs) [2]. The synergistic effect of anti-angiogenesis and immune checkpoint inhibitors has been clarified [3]; VEGF-A, mainly produced by tumor cells and tumor-associated macrophages (TAM), can directly increase the recruitment of Treg and the secretion of immunosuppressive cytokines [4, 5]. VEGF-A could also increase the expression of Fas ligand in tumor endothelial cells, which is associated with low T cell infiltration and Treg dominance [6]. Therefore, anti-VEGF-A therapy synergistically interacts with ICIs by normalizing blood vessels, modulating the immune microenvironment and increasing intratumoral invasion and the survival of cytotoxic T cells.

Cytokines are secreted by tumor cells and stromal cells in the tumor microenvironment (TME), and homing cells such as lymphocytes, macrophages, platelets, etc. are recruited [7–9]. Complex cytokine and chemokine pathway networks accelerate tumor growth and metastasis, and play an important role in the chemotaxis of macrophages, lymphocytes and fibroblasts in cancer. The inflammatory and hypoxic TME produces a large number of chemokines that recruit specific cells through blood vessels [10]. In order to overcome these difficulties in clinical application, biomimetic techniques, including modifying the surface of nanoparticles with various cell membrane proteins [11, 12] which participates in binding to tumor cells and strengthen tumor target, have been introduced to give new opportunities to reinforce biocompatibility and improve therapeutic effectiveness [13]. Among the current bionic carriers, platelets exhibit some special properties. Firstly, it has received widespread attention for its recognition and interaction with tumor cells. Secondly, platelet biomimetic nanoparticles have the ability to evade phagocytic cell clearance, which can prolong their circulation time in the blood [14, 15]. Based on the above characteristics, platelet membranes are widely used in tumor targeted therapy. For example, the Kings team has constructed a platelet drug delivery system [14], and in vitro experiments have shown that platelets can specifically bind to tumor cells and induce tumor cell apoptosis. In order to avoid multidrug resistance caused by chemotherapy drugs, Rao et al. combined photothermal therapy (PTT) with tumor cell targeted platelets to construct a platelet promoted photothermal tumor treatment system [16]. Therefore, delivering anti-cancer drugs to the tumor area through platelet membrane biomimetic nanocarriers with immune escape ability and tumor targeting function has full potential in targeted therapy of HCC.

Blood components are absorbed by the tumor tissue through the tumor vascular system, while platelets can interact with the TME and particularly accumulate in the location of cancer. In conclusion, we hypothesize that nanovehicles (PLTM-HMSNs) coated by platelet membrane (PLTM) can actively target tumor sites, thereby delivering anticancer drugs to the most active areas. To prove our hypothesis, we loaded two anticancer drugs, multi-kinase inhibitor (Sorafenib,Sora) and ICIs (anti-PD-1 antibody,aPD-1), into PLTM-HMSNs (aPD-1-PLTM-@HMSNs@Sora); simultaneously, the influence of TME on platelet biomimetic nanomaterials in HCC was studied, and the mechanism of some biomimetic nanoparticles effectively killing tumor cells was revealed.

## Materials and methods

### Cell culture and tumor models

MHCC97H and HepG2 cell lines were purchased from Shanghai Zhongqiao Xinzhou Biology Co, LTD. Mouse liver cancer cell line H22 was donated by Jin Junpei’s team from Xi ‘an Jiaotong University. The H22 cell line was passed in vivo and used in the third passage. Male BALB/c mice aged 4 weeks, were bought from the Animal Experimental Center of Tongji University, and housed in tongji University’s SPF level laboratory animal room. Tongji University’s guidelines for the care and use of laboratory animals were complied with during the whole experiment. 5 × 10^5^ cells were injected into back of each mouse to produce H22 tumors. All animal procedures defer to the agency Animal Use and Care Committee and are conducted in an ethical and humane manner.

### Materials

N-hydroxysuccinimide (NHS), 2-(n-morpholine) ethanesulfonic acid (MES), Hexadecyl trimethyl ammonium chloride (CTAC), triethanolamine (TEA) and 3-[2- (2-aminoethyl amino) ethyl amino] propyl trimethoxysilane (APTES, ≧98%) were from Aladin Reagent Co, LTD. Sorafenib (Sora) was obtained from MCE. The anti-PD-1 antibody (aPD-1) used in vivo was purchased from Selleck (cat. no. A2005). CD62P (Biolegend, cat.no.148,305), Foxp3 (abcam, cat.no. ab36607), CD41 (abcam, cat.no.134,131), CD4 (SANTA, cat.no. SC19641), CD8 (SANTA, cat.no. SC1177) were also used.

### Synthesis of hollow mesoporous silicon nanoparticles

The monodisperse silicon dioxide (SiO_2_) particles were synthesized by Stober method [17]. Added 3 mL ethyl orthosilicate (TEOS) and mixed for 6 h to prepare sSiO_2_ - NH_2_. Then, we synthesized sSiO_2_-NH_2_@mSiO_2_: mixed 220 mL water, 10 mL ethanol and 1200 mg CTAB, next the obtained sSiO_2_-NH_2_ nanoparticles were added to the mixture and stirred for 30 min. Then 0.975 mL TEOS and 0.1 mL aminopropyltriethoxysilane were added and stirred at room temperature overnight. Finally, the hollow structure was formed by etching the solid core. At this point, the synthesis of particle sSiO_2_-NH_2_@mSiO_2_ was completed. The synthetic HMSNs particles were dispersed in FITC and reacted to prepare FITC-labeled HMSNs. DLS, SEM and TEM were used for characterization.

### Functionalization of PLTM and HMSNs

Platelet-containing plasma was prepared from EDTA anticoagulant fresh mouse blood by differential centrifugation at 4℃, and was blended with PBS containing 1 mM EDTA, 2 mM prostaglandin E1 and protease inhibitor, then platelets were collected by centrifugation at 4℃ at 800 g for 20 min. PLTM was got from platelet suspension after repeated freeze-thaw cycles and centrifuged at 4000 g for 5 min. Subsequently, PLTM and Sulfo-SMCC were intermingled in PBS at a molar ratio of 1:1.2 at 4℃ for 2 h. The mercapto-activated PLTM was recovered using a filter (molecular weight rejection = 20 kDa). The surface of HMSNs was positive charge. Then PLTM, with negative surface charge, was immobilized on the positively charged particle surface by co-incubating the PLTM extracted from 1mL blood with 2 mg HMSNs.

### Loading Sora and aPD-1

Sora and HMSNs were poured into PBS and stirred for 24 h. Then the mixture was centrifuged and the precipitate was collected for drying. The concentration of Sora in the supernatant was determined by UV-VIS method, and the drug loading of Sora was calculated.

For aPD-1 conjugation, PLTM extracted from 2 mL blood was first re-suspended in PBS (pH = 8, + PGE1, 1 µM) and incubated with 0.1 mg/mL Trauts reagent at room temperature for 30 min; then excessive Trauts reagent was removed by centrifugation and washed 3 times with PBS buffer (+ PGE1, 1 µM); finally, PLTM-HMSNs@Sora and aPD-1 were mixed in PBS buffer (+ PGE1, 1µM) and aPD-1-PLTM-HMSNs@Sora was obtained after centrifugation for 10 min. The amount of aPD-1 conjugated to the platelets was measured via ELISA (Rat IgG Total ELISA Kit; eBioscience). After labelling aPD-1 with CY5.5 and PLTM-HMSNs@Sora with FITC, the conjugation of aPD-1 with PLTM-HMSNs@Sora was verified by confocal microscopy.

### Cell uptake experiment

MHCC97H and HepG2 cells were inoculated into culture plates, and medium containing 20 µg/mL FITC-labeled HMSNs@Sora and aPD-1-PM-HMSNs@Sora was supplied respectively 24 h later. The cell nucleus and membrane were labeled with Hoescht 333,342 and DIL respectively, and then the distribution of particles in HCC cells was observed. Flow cytometry was used to quantify NP uptake by MHCC97H cells and HepG2 cells.

### Biosafety experiment

CCK-8 assay was used to test cytotoxicity and the absorbance (OD) was examined with a microplate reader at 450 nm. MHCC97H cells and HepG2 cells were inoculated in 96-well plates at a density of 1 × 10^5^ cells per well for 24 h. Then, the same concentration of HMSNs and PM-HMSNS dispersions were added respectively.

Automated hematology analyzer and hematoxylin–eosin (HE) staining were used to verify the experimental safety in vivo. BALB/c mice were injected with nanoparticles by tail vein at the following dose and frequency (0.5 mg each time for three days). Blood was taken from the orbit to monitor hepatorenal toxicity, such as blood urine nitrogen (BUN), alanine aminotransferase (ALT), aspartate aminotransferase (AST), alkaline phosphatase (ALP), and the heart, liver, spleen, lung and kidney of the mice were stained with HE after the mice were sacrificed by cervical dislocation.

### In vivo tumor therapy

Mice with H22 tumor (18–20 g) were randomly divided into 5 groups (*n* = 5) and given PBS, Sora, aPD-1, Sora + aPD-1 and aPD-1-PLTM-HMSNs@Sora, respectively. Tumor volume and diameter were measured and calculated when all disposal was completed.

Furthermore, the tumor was removed from the mice after treatment for HE, immunohistochemistry and immunofluorescence staining. The expression of VEGF-A in the tumor site was observed by immunohistochemistry, and the expression of CD8 + T, CD4 + T and Treg cells was visualized by immunofluorescence.

### Statistical analysis

All statistical analyses were performed using GraphPad Prism 6.0a for Mac OSX (San Diego, CA, USA) and all data was expressed as mean ± standard deviation (SD). Data was tested with One-way ANOVA and *p < 0.05 (two-tailed) was considered to be significant while **p < 0.01 or ***p < 0.001 was regarded as being highly significant.

## Results

### Preparation and characterization of aPD-1-PLTM@HMSNs@Sora

**Fig. 1:**
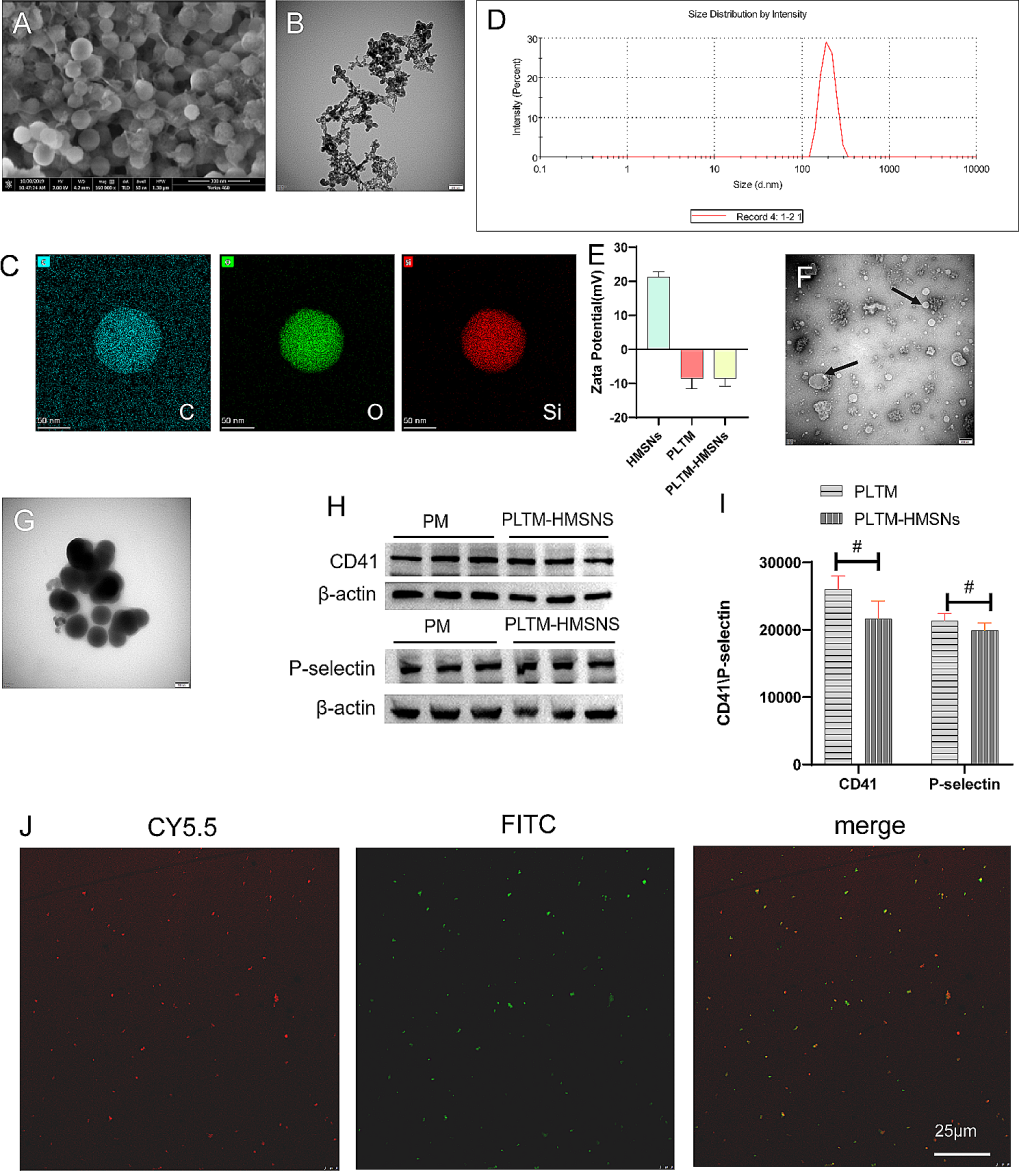
Characterization of nanoparticles. (**A**) SEM images: HMSNs. Scale bar, 300 nm. (**B**) TEM images: HMSNs particles. Scale bar, 200 nm. (**C**) The surface element (**C**, O, Si) mapping of HMSNs. (**D**) HMSNs particles size distribution by intensity. (**E**) Zeta potential of HMSNs (-NH_2_), PLTM and PLTM-HMSNs (mean ± SD; *n* = 3). (**F**, **G**) TEM images: PLTM, Scale bar, 200 nm; PLTM-HMSNs, Scale bar, 100 nm. (**H**) Membrane proteins detected by western blot. (**H**, **I**) CD41 and P-selectin during the construction of PLTM-HMSNs, PLTM as a control (mean ± SD). (**J**) Confocal laser photo of aPD-1-PLTM-HMSNs@Sora (CY5.5 (red) stained aPD-1, FITC (green) labeled HMSNs)

HMSNs was synthesized and modified by amino group, and Sora was loaded into HMSNs through physical methods. HMSNs was characterized by SEM and TEM. The particle size of HMSNs was uniform under SEM microscope, and the ordered microporous structure was displayed by TEM image (Fig. 1A, B). The element mapping of HMSNs showed that the C, O and Si elements were distributed in the structure (Fig. 1C). Thanks to the rich porosity and large pore diameter of HMSNs (Fig. 1D), Sora encapsulation is readily accessible to HMSNs (Supplementary Fig. 1). The size, Zeta potential, drug loading efficiency and encapsulation efficiency of all nanoparticles are all listed in Table 1.

**Table 1:** Characterization properties of blank NPs and drug-loaded NPs

Nanoparticles	Diameter (nm)	Zeta potential (mV)	Ecapsulation efficiency (%)	Drug loading (%)
Platelets	1568.33 ± 652.62	-8.61 ± 2.89	-	-
HMSNs	250.57 ± 24.16	+ 21.33 ± 1.46	-	-
PLTM-HMSNs@Sora	1069.33 ± 41.86	-8.46 ± 2.41	52.99 ± 1.90%	20.94 ± 0.60%

Secondly, PLTM and HMSNs@Sora were fused under ultrasound to produce PLTM-HMSNs@Sora. The Zeta potential is -8.61 ± 2.89, which is approaching the surface charge of platelets. Contrarily, the surface potential of HMSNs particles coated with PLTM changed from 21.33 ± 1.46 to -8.46 ± 2.41, which was close to the surface potential of PLTM and platelets (*p* = 0.170), and there was no distinct difference between them. The measurement of Zeta potential revealed that the surface charge of different nanoparticles was significantly different (Fig. 1E), which further proved the successful formation of PLTM-HMSNs@Sora. Moreover, the average diameter of platelet vesicles was about 100 nm and TEM images demonstrated that PLTM had a hollow vesicle structure (Fig. 1F) while PLTM-HMSNs had a film on its surface, which is larger than the uncoated HMSNs (Fig. 1G). Fusion between adjacent protein lipid plaques and lipid vesicles may result in complete particle surface coverage.

Platelet membrane protein is the key to the targeted adhesion of platelets to tumor cells [18]. PLTM-HMSNs were incubated in fetal bovine serum at room temperature for 24 h, then platelet membrane proteins were stripped for Western blot analysis. CD41, a platelet-specific expression protein, was used to estimate the amount of PLTM (Fig. 1H), and no significant difference was found in the amount of PLTM coated on particles compared with that of activated platelets (*p* = 0.506). P-selectin is detected to be a glycoprotein expressing on the surface of activated platelets, which mainly partakes in the coaction of both platelets and tumor microenvironment, and specifically aggregates in tumor area. Platelet membrane-associated protein P-selectin was analyzed by Western blot (Fig. 1I) and results certified that there was no marked difference in the amount of P-selectin coated on the particles in comparison with that of activated platelets (*p* = 0.13). These results indicated that platelet membrane proteins remain stable on PM-HMSNs for at least 24 h.

In our study, maleimide linkers was used to facilitate the coupling of aPD-1 with PLTM-HMSNs@Sora [14]. The amount of aPD-1 conjugated to the platelet membrane was measured via ELISA (Rat IgG Total ELISA Kit; eBioscience), and the conjugation rate of a-PD-1 was about 62.5%. The coupling of aPD-1 with PLTM-HMSNs@Sora was observed under laser confocal microscopy at 24 h, and images illustrated that they were stably coupled (Fig. 1J). The results of the above experiments revealed that aPD-1-PLTM-HMSNs@Sora is stable in vitro. We dissolved aPD-1-PLPM-HMSNs@Sora in 10% serum to simulate its stability in vivo. Centrifugal precipitation and resuspension after 24 h, Zeta potential and particle size distribution of aPD-1-PLTM-HMSNs@Sora were measured on Zetasizer Nanoseries. Zata potential is -11.9 mV, particle size is 1103 nm, were similar to the aPD-1-PLTM-HMSNs@Sora. The above results demonstrated that aPD-1-PLTM-HMSNs@Sora had good stability in vivo.

### Cell uptake experiment in vitro

**Fig. 2:**
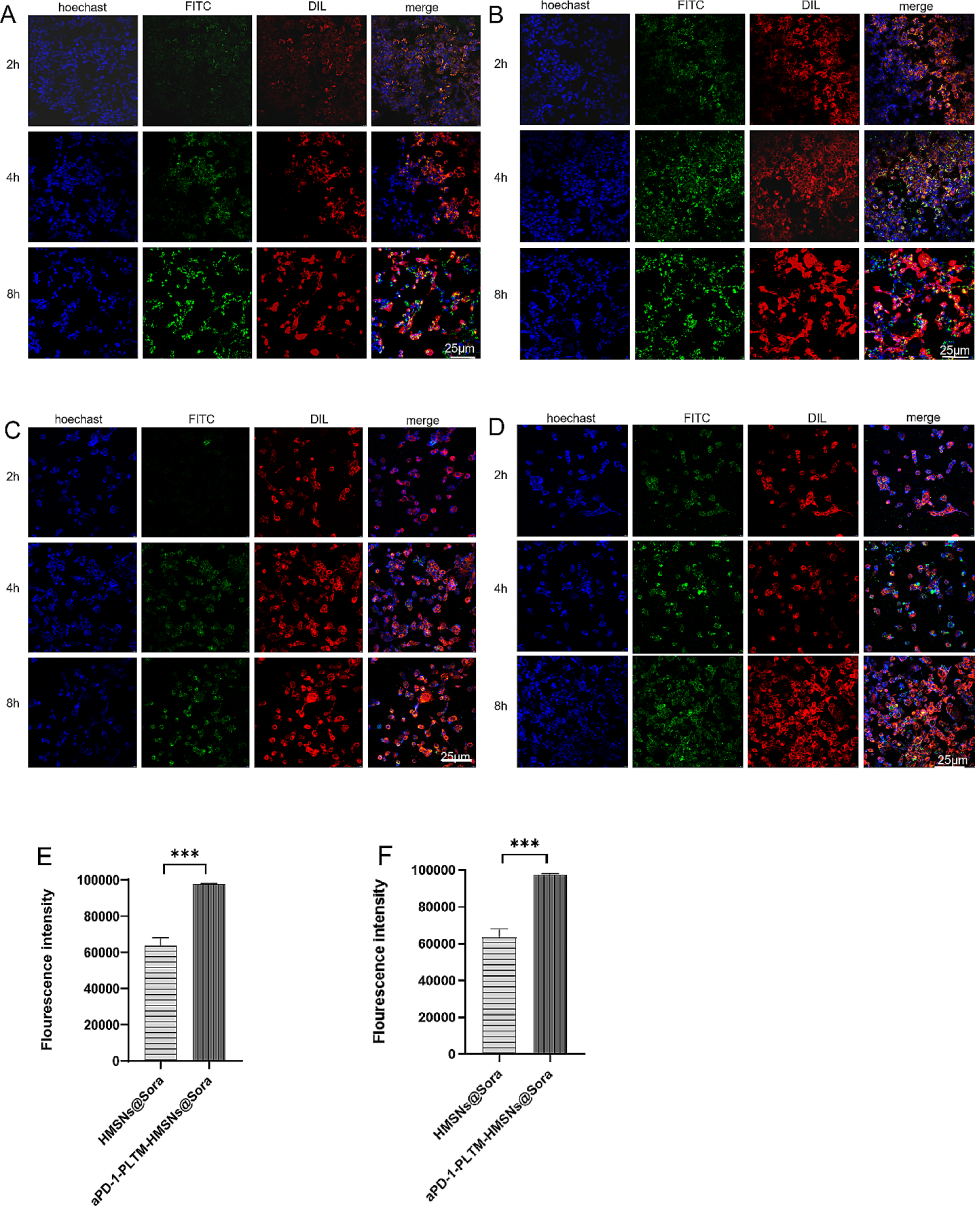
(**A**, **B**) CLSM images of HMSNs@Sora and aPD-1-PLTM-HMSNs@Sora uptaken by MHCC97H cells incubated with FITC-labelled HMSNs for different times. (**C**, **D**) CLSM images of HMSNs@Sora and aPD-1-PLTM-HMSNs@Sora uptaken by Hep-G2 cells incubated with FITC-labelled HMSNs for different times. (**E**, **F**) Corresponding quantified fluorescence intensity for MGCC97H and HepG2 cells. NPs, cell membrane and nucleus were stained with FITC (green), Dil (red) and Hoechst (blue), respectively. Scale bar in the last image can be applied to the others. Scale bar, 25 μm

Cellular uptake is an important basis for nanodelivery drugs. Studies have revealed that platelet membranes potentiate drug-carrying particles the ability to be taken up by tumors and to target tumor cells [19, 20]. We further investigated the interactivity between PLTM - coated nanoparticles and hepatocellular carcinoma cells. HMSNs@Sora and aPD-1-PLTM-HMSNs@Sora at the same concentration were added into MHCC97H and HepG2 cells respectively, and the biological distribution of drug-loaded particles in MHCC97H cells and HepG2 cells was both observed by laser confocal microscopy at 2, 4 and 8 h. It was substantiated that the fluorescence intensity of aPD-1-PLTM-HMSNs@Sora grew continuously and was higher than that of HMSNs@Sora from 2 h in MHCC97H (Fig. 2A, B). Additionally, it was found that the fluorescence intensity of HMSNs@Sora in HepG2 cells was lower than that of aPD-1-PLTM-HMSNs@Sora at 4 h while that of aPD-1-PLTM-HMSNs@Sora continued to rise and was still going up at 8 h (Fig. 2C, D).

In order to further evaluate the endocytosis mechanism of cellular uptake, flow cytometry (Fig. 2E, F) demonstrated that the cellular uptake of aPD-1-PLTM-HMSNs@Sora by MGCC97H and HepG2 cells was significantly greater than that of HMSNs@Sora at 8 h, respectively. All above results strongly suggested that aPD-1-PM-HMSNs@Sora could delay the endocytosis rate of the nanodrug delivery system, and the fluorescence was enhanced with the extension of time, providing a new direction for the accumulation of intracellular drug concentration and the improvement of tumor killing effect.

### Biosafety of vector PLTM-HMSNS particles

**Fig. 3:**
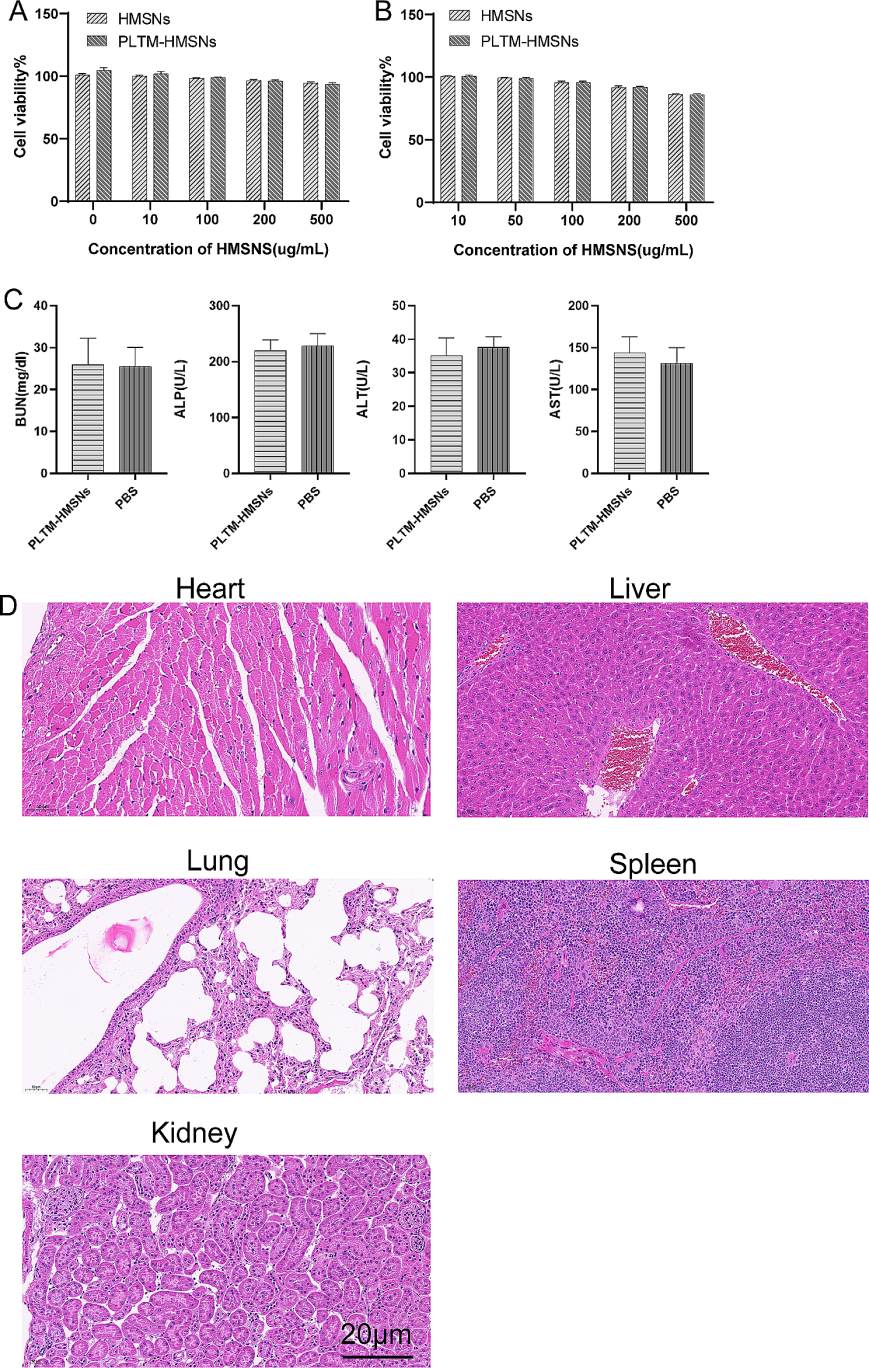
Cell viability of MHCC97H (**A**) and HepG2 (**B**) after incubation with different concentrations of HMSNs and PLTM-HMSNs for 24 h. (**C**) The liver and kidney function indexes of BALB/c mice. All results are presented as the mean ± SD. (**D**) HE-stained organs from tumor-free BALB/c mice after injected with nanoparticles. Scale bar, 20 μm

Safe and effective carrier system is the premise of successful drug combination therapy. Cytotoxicity is an important factor for HMSNs or PLTM-HMSNs as contrast agents in vivo. CCK8 assay was used to detect the cell viability after 24 h incubation with HMSNs or PLTM-HMSNS. We examined the toxicity of 0-500ug/mL nanocarriers to cells. Even when the concentration of HMSNs or PLTM-HMSNS reached 500 µg/mL, the survival rate of MHCC97H cells was respectively 95.53% and 93.84% while that of HepG2 cells was 88.36% and 88.78%, respectively (Fig. 3A, B). The results corroborated that PLTM-HMSNs had no cytotoxic effect on cells and that PLTM-HMSNS was a drug release system with high biocompatibility.

To evaluate the biosafety of PLTM-HMSNs in vivo, liver and kidney toxicity were tested in tumor-free BALB/c mice which were injected with nanoparticles by tail vein. After 30 days, liver and kidney toxicity were detected via cardiac blood sampling (Fig. 3C). AST (*p* = 0.264), ALT (*p* = O.472), ALP (*p* = 0.961) and BUN (*p* = 0.0.705) were not uncovered to be strikingly different compared with the control group. The above results confirmed that there was no abnormality in liver and kidney function in mice injected with PLTM-HMSNS. When the mice were sacrificed, important organs were also taken for detection, and no necrotic area was observed under the microscope (Fig. 3D).

### Antitumor effect of drug-loaded PLTM-HMSNS

**Fig. 4:**
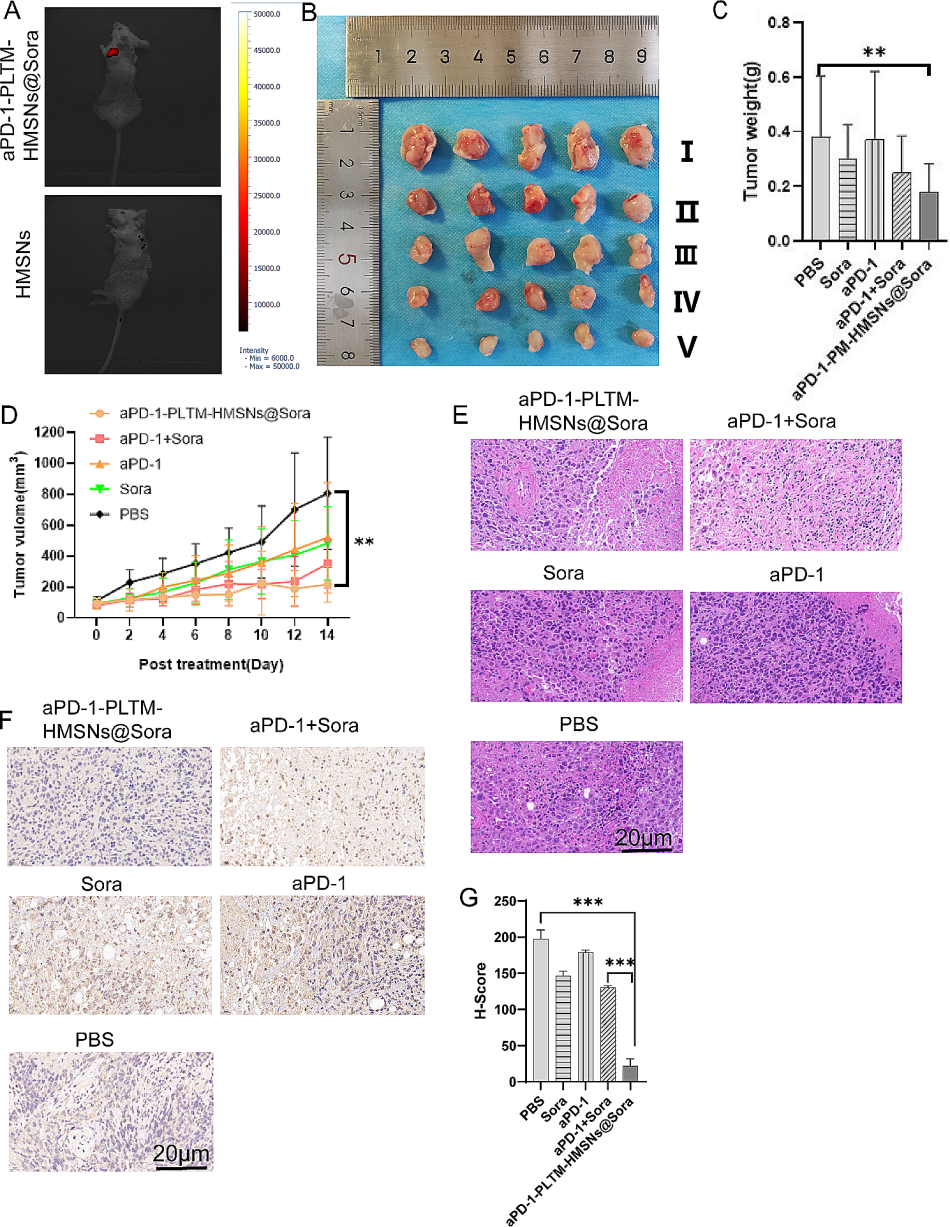
Anti-tumor efficacy assays in vivo of tumor-bearing mice. (**A**) In Vivo fluorescence imaging of Cy5.5 labeled aPD-1-PLTM-HMSNs@Sora in H22 tumor-bearing mice. (**B**) Tumor macroscopic images (1: PBS; 2: Sora; 3: aPD-1; 4: aPD-1 + Sora; 5: aPD-1-PLTM-HMSNs@Sora). (**C**) Tumor weight. (**D**) Growth curves of tumors in each group. Error bars represent the SD (*n* = 5). (**E**) HE stained tumor slices in each treatment group. (**F**) Expression of VEGFR2 in tumor tissue. (**G**) Quantitative analysis of the inhibition by Sora, aPD-1, aPD-1 + Sora, aPD-1-PLTM-HMSNs@Sora on the VEGF-A of tumor (H-score means Histochemistry score). Scale bar, 20 μm. Error bars represent the SD (*n* = 3)

The antitumor effect in vivo was evaluated using H22 tumor-bearing BALB/c mice. The tumor targeting ability of Cy5.5 labeled PD-1-PLTM-HMSNs@Sora and Cy5.5 labeled HMSNs was evaluated by in vivo fluorescence imaging. Strong fluorescence signals were observed at the tumor site 12 h after injection of the aPD-1-PLTM-HMSNs@Sora (Fig. 4A), demonstrating the targeting ability of the biomimetic nanoparticles. In contrast, uncoated Cy5.5 labeled HMSNs observed low fluorescence signals at the tumor site throughout the treatment period.

Tumor volume and weight of each group were recorded during the research process, tumor photos were taken at the end of this stage (Fig. 4B) and tumor weight was weighed. Although aPD-1, Sora, aPD-1 + Sora and aPD-1-PLTM-HMSNs@Sora all inhibited tumor growth to a certain extent, aPD-1-PLTM-HMSNs@Sora produced the most significant inhibitory effect. We intervened under the same conditions in female tumor bearing BALB/c mice, and the treatment results were similar to those in male tumor bearing BALB/c mice (Figure S2). Figure 4C and D illustrated that the aPD-1-PLTM-HMSNs@Sora group had a higher tumor inhibition rate than the aPD-1, Sora and aPD-1 + Sora groups, and the inhibitory effect on tumor growth was ranked as aPD-1-PLTM-HMSNs@Sora > aPD-1 + Sora > aPD-1 > Sora. According to Fig. 4D, the average tumor volumes of mice in aPD-1-PLTM-HMSNs@Sora, aPD-1 + Sora, aPD-1 and Sora group were respectively 26.85%, 39.03%, 64.45% and 59.89% of those of PBS group at the day 14th. In contrast with the aPD-1 + Sora group, inhibition of tumor growth was more prominent in the aPD-1-PLTM-HMSNs@Sora group, which may benefit from PM-mediated targeted tumor cell delivery. Finally, the body weight of the mice receiving different drugs remained stable during the research stage.

The antitumor function of aPD-1-PLTM-HMSNs@Sora in vivo was determined by histopathological analysis. HE staining of tumor tissues testified that the nuclei of tumor treated with aPD-1-PLTM-HMSNs@Sora were destroyed obviously, however, this condition was less prevalent in the group supplied aPD-1 + Sora (Fig. 4E), suggesting that aPD-1-PLTM-HMSNs@Sora contributed to more effective inhibition of tumor growth. In addition, aPD-1-PLTM-HMSNs@Sora inhibited VEGF-A (promoting tumor growth and angiogenesis) expression more efficiently than other treatments which was proved by immunohistochemical data (Fig. 4F, G). Meanwhile, this phenomenon might also partly account for the noteworthy inhibitory effect of aPD-1-PM-HMSNs@Sora on H22 tumor growth.

### T cell mediated immune response

**Fig. 5:**
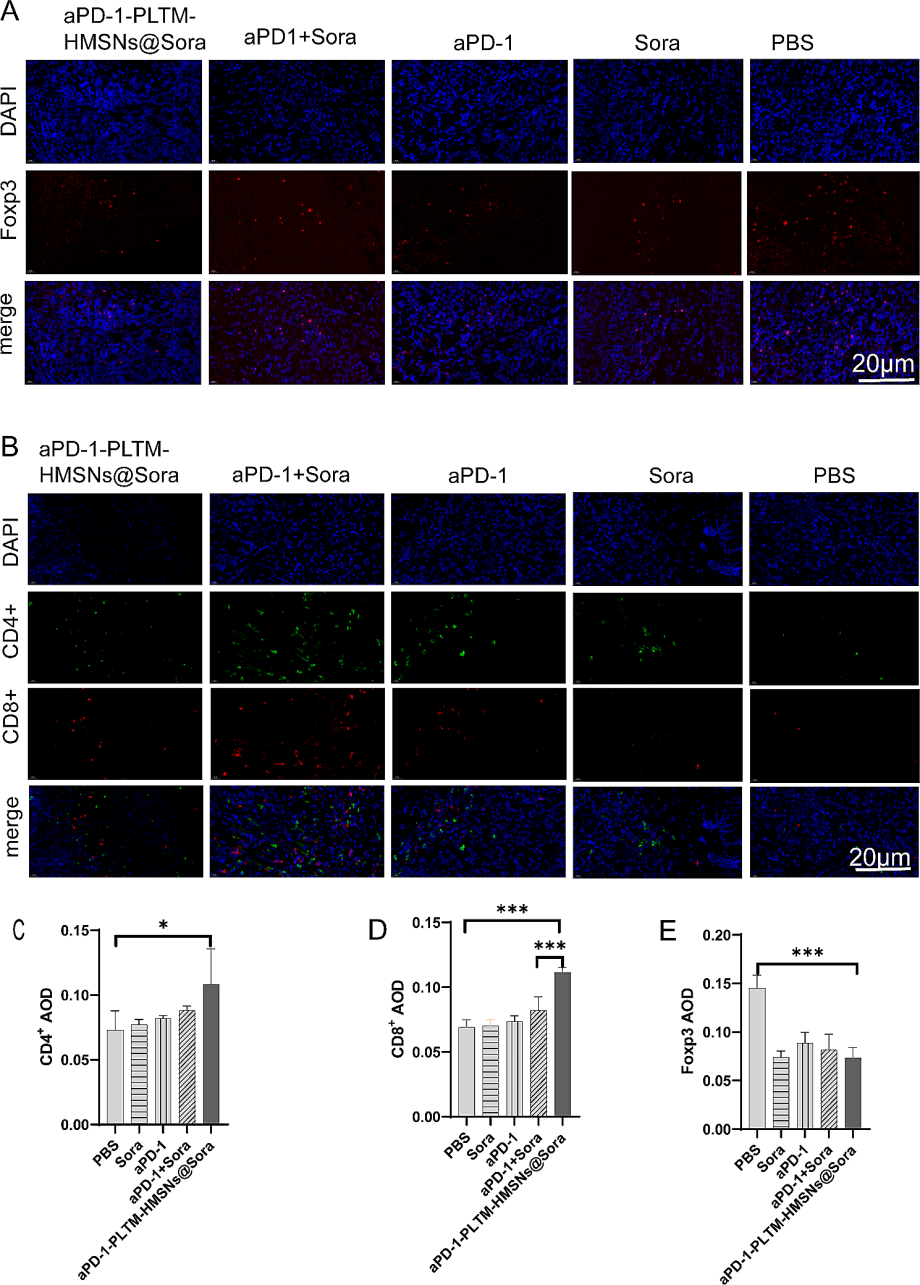
aPD-1-PLTM-HMSNs@Sora triggered a robust, T-cell-mediated anti-tumor immune response. (**A**) Immunofluorescence of residual tumors showed CD4^+^ T cells and CD8^+^ T cells infiltration. Scale bar, 20 μm. (**B**, **C**) Quantitative analysis of the number of CD8^+^ T cells and CD4^+^ effector T cells of tumor after treatment. (**D**) Immunofluorescence of residual tumors showed Foxp3^+^ T cells infiltration. Scale bar, 20 μm (AOD means average optical density). (**E**) Quantitative analysis of the number of Foxp3^+^ T cells of tumor after treatment. Error bars represent the SD (*n* = 3). Statistical significance was calculated via one-way ANOVA with a Tukey post-hoc test. *p < 0.05; **p < 0.01; ***p < 0.001

Subcutaneous tumors were collected on day 14th and the infiltrating lymphocytes were analyzed by immunofluorescence. Immunofluorescence staining demonstrated T cell infiltration in tumor microenvironment was restricted in control group. In contrast, the TME of aPD-1-PLTM-HMSNs@Sora treated mice was highly infiltrated with CD8^+^ and CD4^+^ T cells (Fig. 5A). The growth rate of tumor in mice being injected aPD-1-PLTM-HMSNs@Sora was slow (Fig. 4B), which might be associated with a markedly growth of the absolute number of CD4^+^ and CD8^+^ T cells in TME (Fig. 5B, C). More importantly, the absolute quantity of CD4^+^ and CD8^+^ T cells in lung tumors of mice dealt with aPD-1-PLTM-HMSNs@Sora increased by 1.5 times compared with those of the control group, while those in mice being disposed with aPD1 + Sora only increased by 20%. The average number of CD4^+^ and CD8^+^ T cells in aPD-1-PLTM-HMSNs@Sora, aPD-1 + Sora, aPD-1, Sora group were 148.49%/166.96%, 120.52%/118.93%, 118.89%/106.59% and 105.48%/102.14% of those in PBS group, respectively. As shown in Fig. 4C, tumor weight of mice treated with aPD-1-PLTM-HMSNs@Sora was dramatically lighter than that of the aPD-1 + Sora group, which validated that aPD-1-PLTM-HMSNs@Sora therapy could promote the anti-tumor effect of TME immune cells. The number of infiltrating CD4^+^ Foxp3^+^ T cells in TME was also detected. (Fig. 5D). As illustrated in Fig. 5E, the number of Tregs in the TME of mice administered aPD-1-PLTM-HMSNs@Sora was significantly reduced in comparison with that of aPD-1 + Sora, which may be related to the inhibition of VEGF-A production by high accumulation of Sora in local tumor areas (Fig. 4E). To summarize, aPD-1-PLTM-HMSNs@Sora had the strongest synergistic antitumor effect and could directionally deliver aPD-1 and Sora to its most active destination, consequently regulating the tumor immune microenvironment and stimulating T cell-mediated antitumor immune response.

## Discussion

At present, most nanoparticles mainly rely on the chemical modification of specific targeted parts to obtain the targeting ability [21], but nanosystems accumulate at the tumor target in insufficient quantities after intravenous injection because of shielding by rapid immune clearance by the mononuclear phagocyte system [22–24]. Biomimetic strategy can maintain the stable and uniform expression of natural targeting ligands, and has higher corresponding tumor-targeting efficacy. This strategy retains platelet membrane accessibility and unique physiological functions, such as vascular injury response, recognition and interaction with circulating tumor cells [15, 25–28], and can avoid immune clearance. Biomimetic strategies can reduce the adsorption of nonspecific proteins in vivo and prevent the degradation caused by plasma protease [29]. P-selectin mediates the interaction between platelets and tumor cells. The mechanism underlying this specific aggregation includes P-Selectin and CD44 receptors [30], and structure-based capture [31, 32] are significantly different from other biomimetic carriers derived from, for instance, cancer or blood cell. The mechanism of decreased therapeutic effect of anti-PD-1 / PD-L1 may be that the antibody binds to normal tissue when injected intravenously [33, 34]. Therefore, the ideal administration strategy of immune checkpoint inhibitors for anti-tumor is to maximize the concentration of antibodies in the lesion site. In this study, the therapeutic effect of biomimetic strategy was significantly better than aPD-1 + Sora or single drug therapy. Compared with the existing immunotherapy, platelets targeting tumor microenvironment strategy can overcome several difficulties in tumor immunotherapy. Platelet-biomimetic nanoparticles often display elongated blood circulation times and decreased absorption by healthy organs [35–37]. Therefore, platelet-derived nanocarriers with immune-evading and tumor-targeting capabilities are expected to reach their full potential in immune therapy for liver cancer by maximizing the delivery anti-cancer drugs to the tumor target.

Sorafenib (Sora) has a wide range of anti-tumor effects, but for some patients, long-term administration of Sora alone is prone to drug resistance [38, 39]. At the same time, because of its unique adverse toxic effects, patients cannot continue to receive treatment, which makes many researchers reduce the dose of Sora by combining Sora with other drugs [40, 41]. In recent studies, the combination of targeted therapy and immunotherapy has shown a synergistic effect, promoting the treatment of advanced liver cancer into a new era. In our study, Sora or aPD-1 was loaded into PLTM-HMSNs nanoparticles to improve the therapeutic effect of liver cancer, which has not been reported before. Antiangiogenic drugs up-regulate the migration and function of T cells, reverse the expression of immunosuppressive cells caused by tissue hypoxia, and regulate the immune microenvironment. When combined with immune checkpoint inhibitors in the treatment of HCC, a positive feedback loop between vascular normalization and immune reconstruction can be established, and the phenomenon of greater tumor regression and higher efficiency can be seen in mouse experiments [42, 43].

Consistently, we found that aPD-1-PLTM-HMSNs @ Sora could significantly inhibit the expression of VEGFR2 (Fig. 4F). The high infiltration of effector T cells in tumor further confirmed that aPD-1-PLTM-HMSNs @Sora had enhanced anti-tumor effect. This study shows that aPD-1-PLTM-HMSNs @ Sora has obvious inhibitory and therapeutic effects on liver cancer, and has high clinical application value.

## Conclusions

In this study, we successfully designed a platelet nanocomplex with comparable biocompatibility for continuous and directional delivery of aPD-1 and Sora, which could triumphantly inhibit tumor growth through targeting of tumor cells and vessels. Utilizing the specific interaction between platelets and cancer cells, PLTM-HMSNs can effectively transfer aPD-1 and Sora into TAM, inhibit the production of Treg chemokines, and activate the anti-tumor immune response mediated by T cells. Besides, PLTM-HMSNs can be digested after being took into cancer cells, then may enhance Sora accumulation in the cells and activate endogenous apoptotic pathways. aPD-1-PLTM-HMSNs@Sora has been confirmed to have a good synergistic antitumor effect. On account of its unique bionic characteristics, good targeted property and multi-target and comprehensive therapeutic effect, this PLTM-HMSNs can also be combined with other anti-tumor drugs to deliver them on corresponding targets of tumor cells, thus achieving synergistic anti-tumor effect. Lastly, these PLTM-HMSNs could also be further used to prevent metastasis of cancer cells since metastatic cancer cells can bind specifically to platelets to escape clearance by the immune system and to spread to new tissues [44].

### Electronic supplementary material

Below is the link to the electronic supplementary material.

Supplementary Material 1

Supplementary Material 2

## Data Availability

The datasets used and/or analysed during the current study are available from the corresponding author on reasonable request.
