# Supplementary figures and images for: Inhibition of growth of hepatocellular carcinoma by co-delivery of anti-PD-1 antibody and sorafenib using biomimetic nano-platelets
Source: BMC Cancer. 2024 Feb 26;24:273. doi: 10.1186/s12885-024-12006-1 (PMC10898182; doi:10.1186/s12885-024-12006-1)

Gels and blots


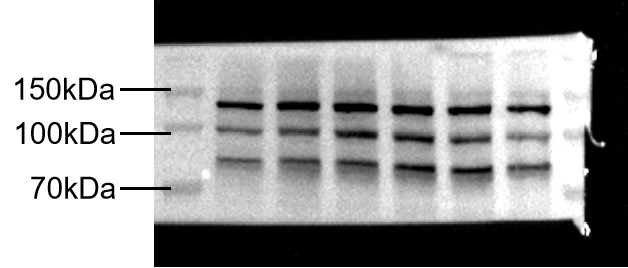


CD41


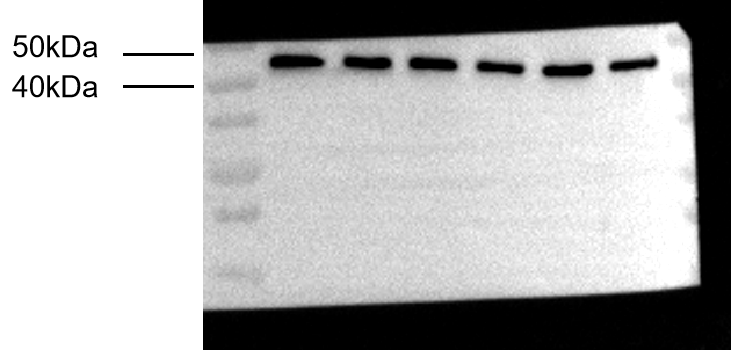


β-actin(CD41)


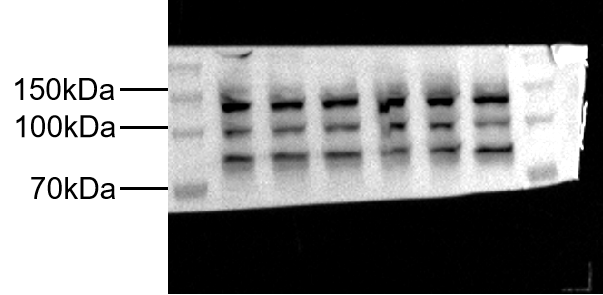


1. Selection


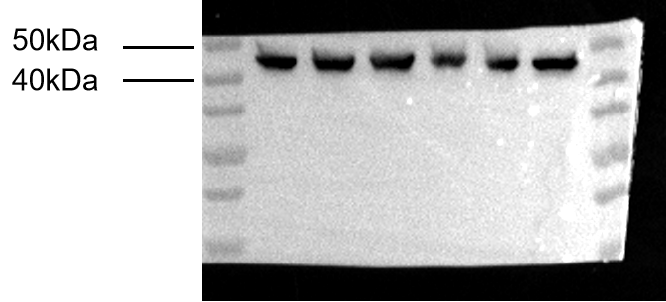


β-actin(P-Selection)

Supplement: Supplementary file 1 — Supplementary Material 1 [file 12885_2024_12006_MOESM1_ESM.docx]
