# Supplementary material for: Inhibition of growth of hepatocellular carcinoma by co-delivery of anti-PD-1 antibody and sorafenib using biomimetic nano-platelets
Source: BMC Cancer. 2024 Feb 26;24:273. doi: 10.1186/s12885-024-12006-1 (PMC10898182; doi:10.1186/s12885-024-12006-1)
Supplement: Supplementary file 2 — Supplementary Material 2 [file 12885_2024_12006_MOESM2_ESM.docx]

**Supplementary Figures**


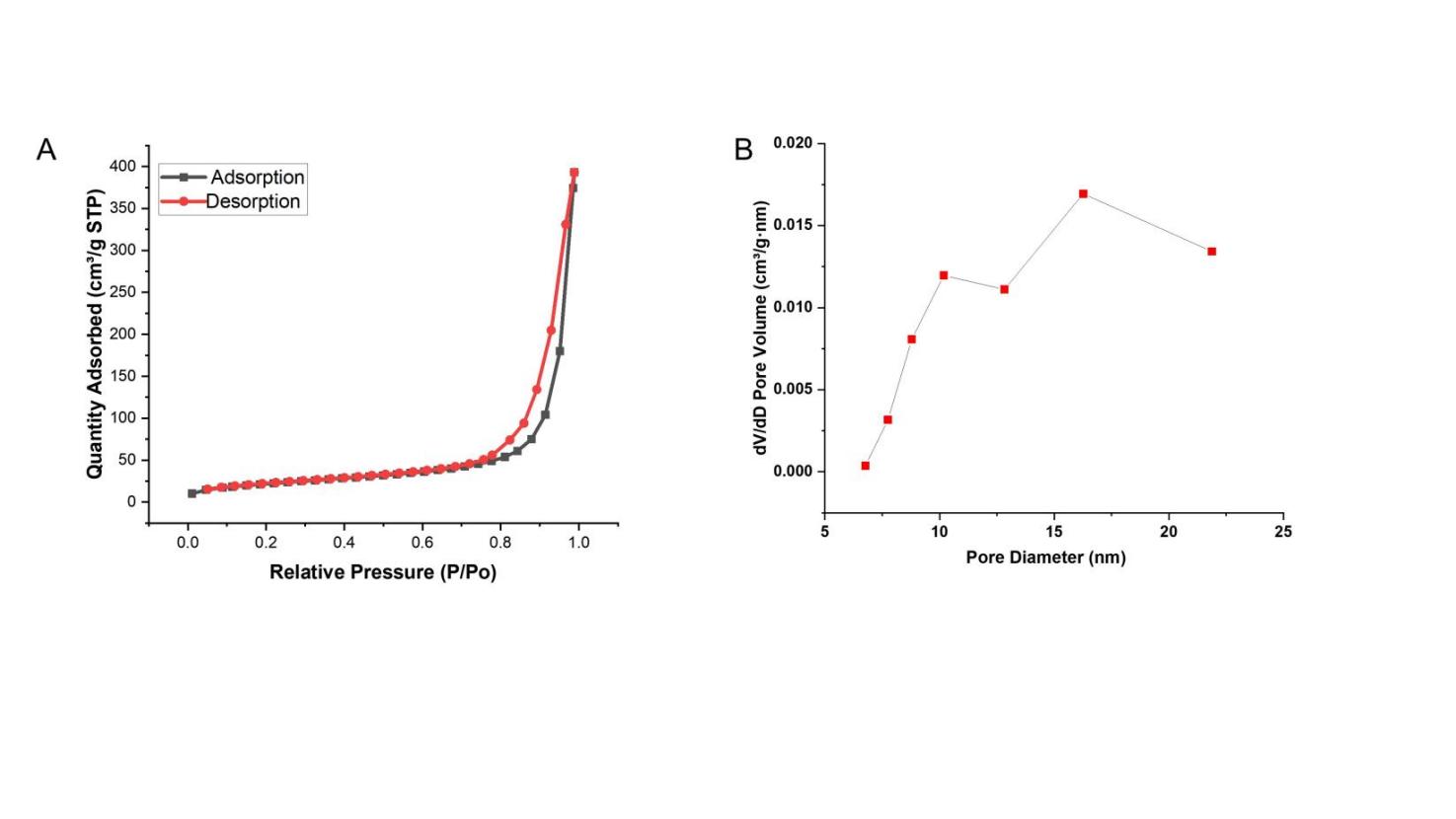


Figure S1 (A) N2 adsorption and desorption isotherms of HMSNs, and (B) Pore diameter distributions of HMSNs.


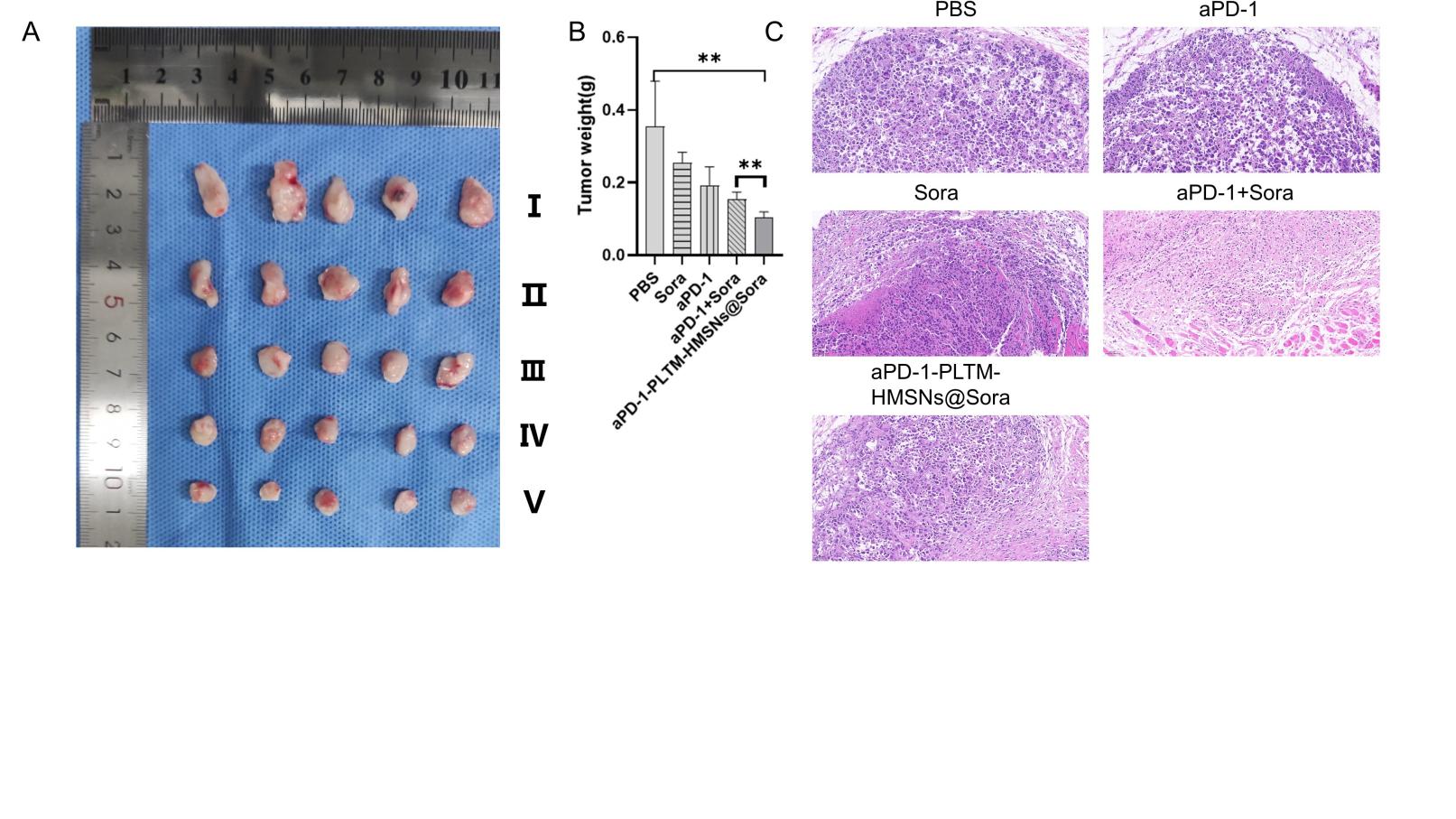


Figure S2 (A) Tumor macroscopic images(1:PBS; 2:Sora; 3: aPD-1; 4:aPD-1+Sora; 5:aPD-1-PLTM-HMSNs@Sora), (B) Tumor weight. Error bars represent the SD. (n= 5), (C)H&E stained tumor slices in each treatment group.
